# Association between meat and fish intake and kidney stone risk: a cross-sectional food frequency questionnaire study in a Chinese population

**DOI:** 10.3389/fnut.2026.1876979

**Published:** 2026-07-22

**Authors:** Xiaowen Zhang, Jianhua Li, Xiangyu Zhang, Haiyan Ju, Shaobin Xia, Ming Chen, Guangyuan Zhang, Peng Han

**Affiliations:** 1 Department of Urology, Southeast University Zhongda Hospital, Nanjing, China; 2 Department of Emergency, Qingdao University Affiliated Hospital, Qingdao, China; 3 Department of Urology, Ulm University Hospital, Ulm, Germany; 4 Department of Urology, Affiliated Hospital of Shandong Second Medical University, Weifang, China; 5 Department of Urology, The First Affiliated Hospital of Nanjing Medical University, Nanjing, China

**Keywords:** Chinese population, food frequency questionnaire, kidney stone, pork consumption, sea fish intake

## Abstract

**Background:**

Dietary factors, particularly meat and fish intake, may influence kidney stone formation, but evidence in the Chinese population is limited.

**Objective:**

To investigate the association between various types of meat and fish consumption and kidney stone prevalence using a food frequency questionnaire (FFQ).

**Methods:**

A cross-sectional study was conducted with 830 participants (299 kidney stone patients, 531 controls) using online and hospital-based FFQs. Logistic regression models assessed associations, adjusting for confounders.

**Results:**

Pork intake showed a non-linear association with kidney stone status. Compared with the lowest intake group, the highest pork intake group was associated with higher odds of kidney stones (OR = 3.321, 95% CI: 1.094–10.044), whereas the highest sea fish intake group was associated with lower odds (OR = 0.331, 95% CI: 0.115–0.936). Processed meat intake was not significantly associated with kidney stone status after adjustment.

**Conclusion:**

Pork consumption is positively associated with kidney stone, and sea fish intake is inversely associated with kidney stone in the Chinese population.

## 1 Introduction

Kidney stones are a common urological condition worldwide and a growing public health concern in China, with a reported prevalence of 10.63% (1). Recurrence rates are high, and stones contribute substantially to healthcare costs and patient morbidity (2).

Dietary habits are a key modifiable risk factor for stone formation. High intake of animal protein and purine-rich foods can increase urinary calcium, uric acid, and oxalate excretion, thereby promoting stone formation (3–5). Conversely, certain nutrients such as omega-3 fatty acids may reduce stone risk by modulating inflammation and urinary metabolites (6).

Previous studies, including large epidemiological cohorts and Mendelian randomization analyses, suggest that oily fish may reduce the risk of kidney stones, while pork and non-oily fish may increase risk (7, 8). However, most of these findings come from Western populations. Given differences in dietary structure and cooking practices, it is important to evaluate these associations in a Chinese context.

This study aimed to assess the relationship between the intake of different types of meat (pork, beef, lamb, poultry, processed meat, organ meat) and fish (oily sea fish vs. non-oily river fish) with kidney stone prevalence in a Chinese population, using a validated simplified FFQ.

## 2 Materials and methods

### 2.1 Study design and population

**Figure 1:**
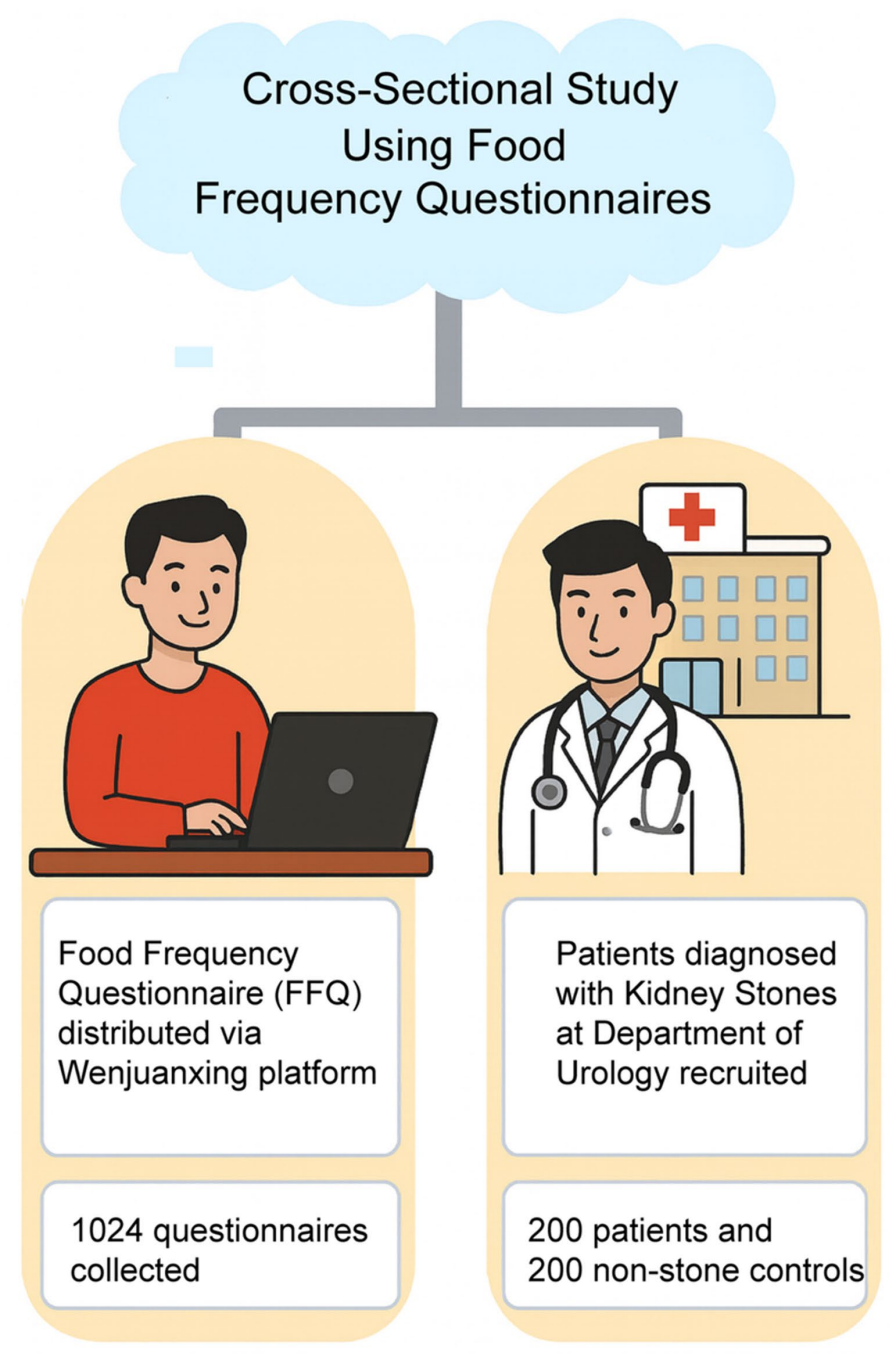
The cross-sectional study design comparing online and hospital-based dietary frequency surveys.

This cross-sectional study was conducted using a dietary frequency questionnaire survey, which consisted of both online and hospital-based components (Figure 1).

For the online survey, a food frequency questionnaire (FFQ) was distributed via the Wenjuanxing platform^1^ between January and March 2024. A total of 1,024 questionnaires were collected, with participants completing the survey voluntarily. Data were retrieved through the platform database.

For the hospital-based survey, 200 patients diagnosed with kidney stones (meeting the inclusion and exclusion criteria) who were admitted to the Department of Urology at Zhongda Hospital from January to December 2023 were recruited, together with 200 non-stone controls attending the hospital during the same period. All participants were informed about the study purpose and voluntarily participated. Given the minimal risk of the study, written informed consent was waived, and the study protocol was approved by the Ethics Committee of Zhongda Hospital (Approval No. 2024ZDSYLL518-P01).

### 2.2 Sample size estimation

Based on the latest epidemiological studies and meta-analyses, the prevalence of kidney stones in China is approximately 10.63% (1). Using the formula for cross-sectional study sample size:

$$N=u_{\alpha }^{2}\times \frac{\mathrm{PQ}}{d^{2}}$$

where *p* = 0.1063, *Q* = 1 − P, *u_α_* = 1.96 (for α = 0.05), and *d* = 0.05, the minimum required sample size was estimated to be 146. The final sample size in this study exceeded this threshold.

### 2.3 Questionnaire design

The questionnaire was developed based on the 2015 China Chronic Disease and Nutrition Survey (9) and the NHANES dietary survey (10), with modifications to shorten completion time to 5–8 min while maintaining validity and reliability (11, 12). Reliability coefficients ranged from 0.629 to 0.674, the Kaiser–Meyer–Olkin (KMO) coefficient was 0.794, and Bartlett’s test of sphericity was significant (*p* < 0.001), indicating suitability for factor analysis. The questionnaire design and investigator training were guided by experts from Nanjing University of Technology and Southeast University School of Public Health.

The questionnaire included demographic characteristics (sex, age, BMI, education level), comorbidities (kidney stones, hypertension, diabetes), daily water intake, and meat/fish consumption frequency (pork, beef, mutton, poultry, processed meat, animal offal, sea fish, freshwater fish).

Meat consumption frequency was categorized into four levels: Level 1, never; Level 2, occasionally (1–3 times per month); Level 3, regularly (1–4 times per week); and Level 4, frequently (5–6 times per week or more). The same classification was applied to all meat categories.

### 2.4 Statistical analysis

Statistical analyses were performed using IBM SPSS version 27. Categorical variables were compared using the chi-square test, and associations between dietary factors and kidney stone prevalence were assessed with multivariate logistic regression models, adjusted for potential confounders including sex, age, body mass index (BMI), education level, hypertension, diabetes, and daily water intake.

To assess potential effect modification, subgroup interaction analyses were conducted across several baseline characteristics, including sex, age, BMI, education level, hypertension, diabetes status, and daily water intake. Odds ratios (ORs) and 95% confidence intervals (CIs) were estimated within each subgroup, and *p*-values for interaction were calculated to determine whether associations varied significantly across strata.

The predictive performance of the multivariable logistic regression model was evaluated using receiver operating characteristic (ROC) curve analysis. The area under the curve (AUC) was calculated to quantify model discrimination, with higher AUC values indicating better ability to distinguish between stone formers and non-stone controls.

Statistical significance was set at a two-sided *p*-value of <0.05.

### 2.5 Quality control

The online questionnaire included embedded quality-control items, such as “Which type of meat or fish do you consume most frequently?” Questionnaires were excluded if the reported consumption-frequency responses were inconsistent with the corresponding quality-control answers. In addition, online questionnaires were excluded if kidney stone status was unknown, because the primary outcome could not be determined; if hypertension or diabetes status was unknown, because these variables were predefined covariates in the multivariable analysis; or if the respondent was younger than 20 years, to maintain consistency with the predefined adult study population.

Hospital-based questionnaires were administered by uniformly trained investigators, including the study authors and medical staff from the same department. The study purpose and significance were explained in detail to hospitalized participants to improve trust, cooperation, and consistency in data collection. Hospital-based questionnaires were excluded if participants refused or were unable to provide reliable baseline information, or if hypertension or diabetes status was uncertain.

## 3 Results

### 3.1 Participant characteristics

**Figure 2:**
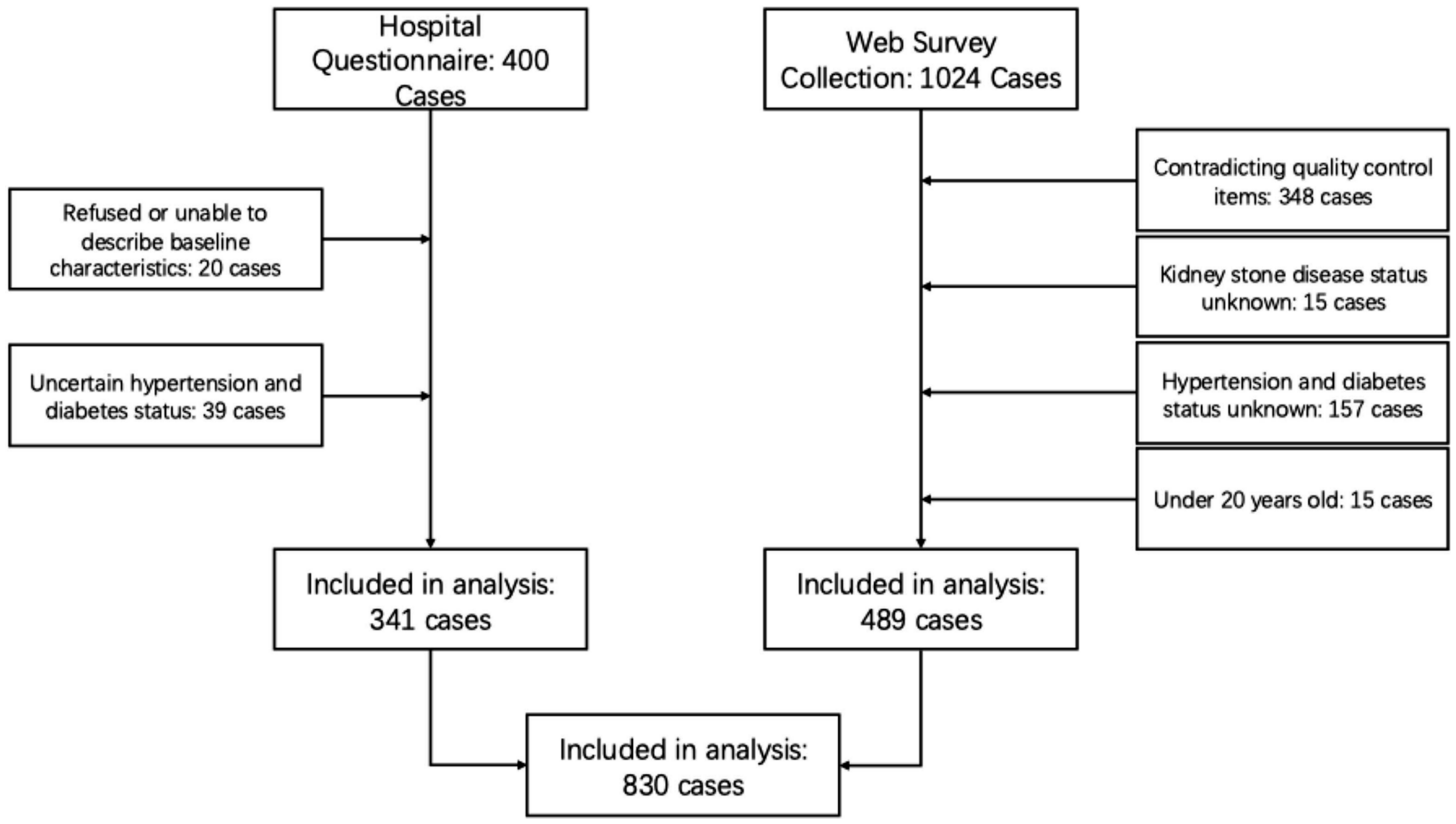
Flow diagram of inclusion/exclusion process and quality control in the questionnaire survey.

A total of 1,424 questionnaires were collected (1,024 online, 400 hospital-based). After quality control, 489 online and 341 hospital-based questionnaires were retained, yielding 830 valid responses for analysis (299 kidney stone patients, 531 controls) (Figure 2).

Demographic characteristics were balanced between groups with respect to sex, age, BMI, and education (*p* > 0.05), while hypertension, diabetes, and lower water intake were significantly more prevalent among kidney stone patients (Table 1).

**Table 1:** Demographic characteristics of questionnaire survey participants.

Variable	Category	Hospital survey			Web survey			Total survey		
		No	Yes	*P*	No	Yes	*P*	No	Yes	*P*
Sex	Female	55	43	0.402	132	43	0.248	187	86	0.065
	Male	123	120		221	93		344	213	
Age (years)	20–30	–	–	0.200	38	16	0.669	38	16	0.627
	31–40	27	25		44	21		71	46	
	41–50	38	47		73	34		111	81	
	51–60	48	50		136	42		184	92	
	>60	60	37		62	23		122	60	
Body mass	Underweight	8	10	0.571	22	9	0.738	30	19	0.379
	Normal	93	74		181	64		274	138	
	Overweight	62	61		121	48		183	109	
	Obese	15	18		29	15		44	33	
Education	Junior high school or below	2	5	0.098	4	2	0.674	6	7	0.608
	High school/Technical school	56	33		58	28		114	61	
	College diploma	28	26		37	15		65	41	
	Bachelor’s degree	61	59		133	53		194	112	
	Master’s degree or above	31	40		121	38		152	78	
Hypertension	No	158	124	0.002	312	101	<0.001	470	225	<0.001*
	Yes	20	39		41	35		61	74	
Diabetes	No	174	142	<0.001	345	122	<0.001	519	264	<0.001*
	Yes	4	21		8	14		12	35	
Daily water intake	No or little extra water	11	28	<0.001	16	21	<0.001	27	49	<0.001*
	400–1,000 mL	36	50		96	45		132	95	
	1,000–2000 mL	70	58		140	52		210	110	
	2000–3,000 ml	48	21		82	13		130	34	
	>3,000 ml	13	6		19	5		32	11	

*Indicates *p* < 0.05, denoting a statistically significant difference.

### 3.2 Meat and fish consumption and kidney stone status

Chi-square analyses showed significant differences between cases and controls in pork (*p* < 0.001), processed meat (*p* < 0.001), and sea fish intake (*p* < 0.001), while no significant associations were observed for beef, mutton, poultry, offal, or freshwater fish (Table 2).

**Table 2:** Univariate analysis of meat intake and kidney stone prevalence among study participants (chi-square test).

Variable	Levels	Non-kidney stone	Kidney stone	*P*
		*N* = 531	*N* = 299	
Pork	1	11	16	<0.001*
	2	370	49	
	3	118	171	
	4	32	63	
Beef	1	17	14	0.212
	2	285	165	
	3	196	94	
	4	33	26	
Mutton	1	49	41	0.142
	2	344	175	
	3	107	61	
	4	31	22	
Poultry	1	7	12	0.076
	2	210	111	
	3	263	143	
	4	51	33	
Processed meat	1	72	50	<0.001
	2	340	141	
	3	98	88	
	4	21	20	
Organ	1	100	65	0.763
	2	336	183	
	3	68	35	
	4	27	16	
River fish	1	49	32	0.705
	2	312	164	
	3	143	85	
	4	27	18	
Sea fish	1	102	101	<0.001
	2	331	150	
	3	75	36	
	4	23	12	

^*^Indicates *P* < 0.05, denoting a statistically significant difference.

**Figure 3:**
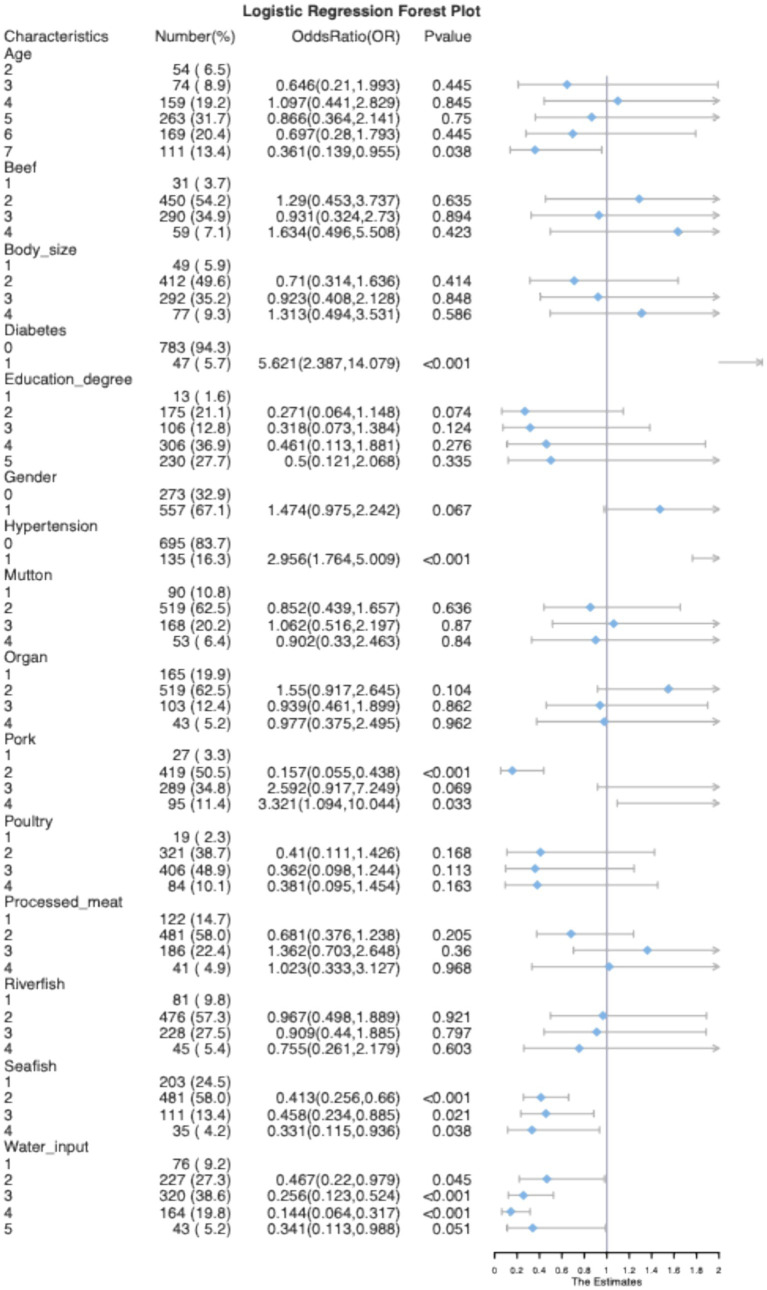
Multivariate logistic regression forest plot.

Multivariable logistic regression analysis (Figure 3) showed a non-linear association between pork consumption frequency and kidney stone status. Compared with Level 1, Level 2 was associated with lower odds of kidney stones (OR = 0.157, 95% CI: 0.055–0.438, *p* < 0.001), whereas Level 4 was associated with higher odds of kidney stones (OR = 3.321, 95% CI: 1.094–10.044, *p* = 0.033). These category-specific findings should be interpreted cautiously and should not be considered evidence of a protective effect at lower consumption levels.

Processed meat consumption was not significantly associated with kidney stone status after multivariable adjustment (*p* > 0.05). Sea fish consumption frequency was inversely associated with kidney stone status. Compared with Level 1, Levels 2, 3, and 4 were associated with lower odds of kidney stones, with ORs of 0.413 (95% CI: 0.256–0.660, *p* < 0.001), 0.458 (95% CI: 0.234–0.885, *p* = 0.021), and 0.331 (95% CI: 0.115–0.936, *p* = 0.038), respectively.

### 3.3 Subgroup analysis

Subgroup interaction analysis was conducted to evaluate potential effect modifiers. No significant interactions were observed for sex (P for interaction = 0.841), age (*p* = 0.256), body mass index categories (*p* = 0.250), diabetes status (*p* = 0.945), or daily water intake (*p* = 0.231). Although males exhibited a slightly elevated risk compared with females (OR = 1.36, 95% CI: 0.998–1.863), the difference was not statistically significant. Similarly, individuals under 60 years had a lower but non-significant risk compared with those aged 60 years and above (OR = 0.80, 95% CI: 0.59–1.09).

**Figure 4:**
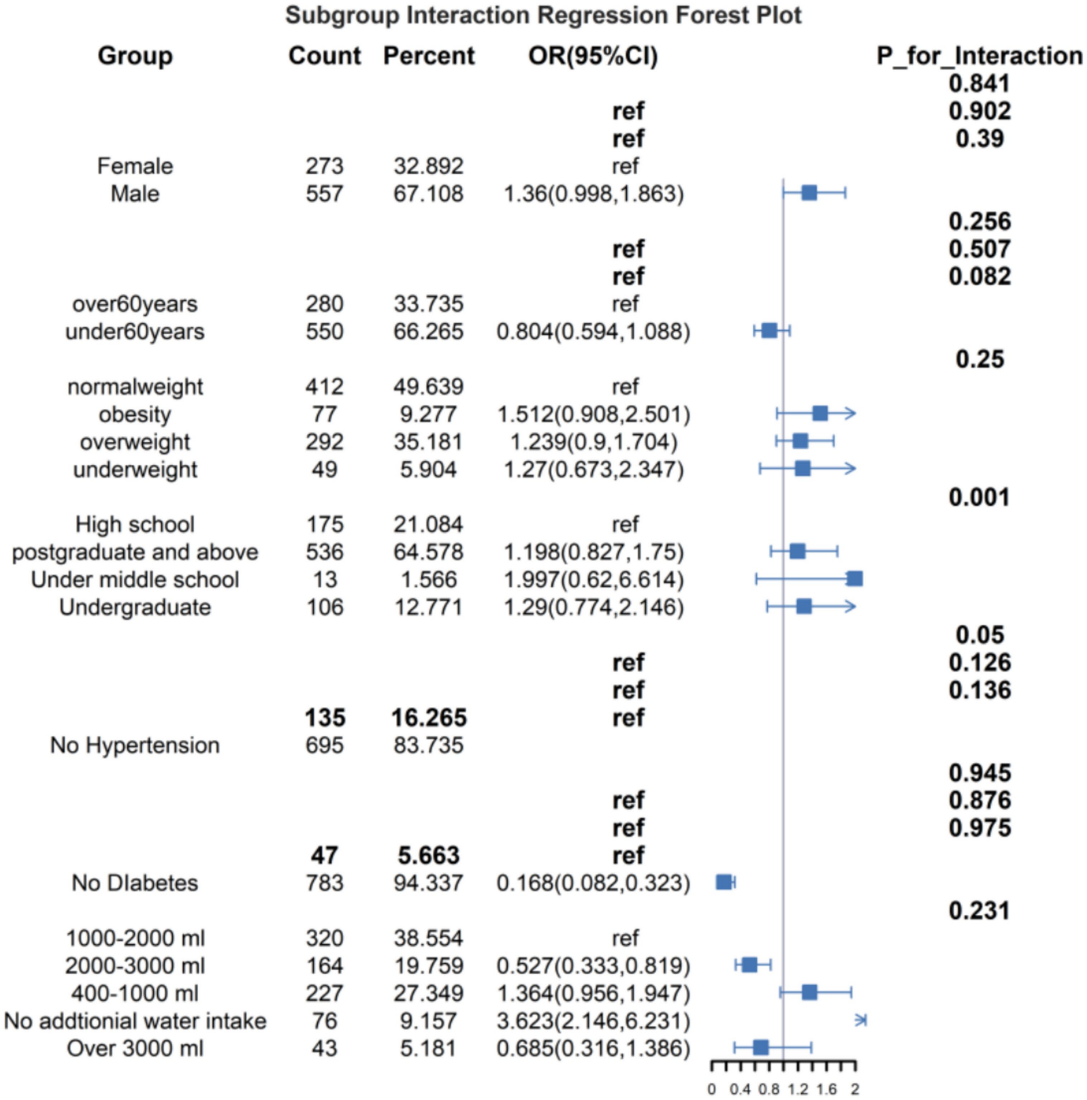
Subgroup analysis of kidney stone risk.

Educational attainment was the only factor showing a statistically significant interaction (*P* for interaction = 0.001), suggesting that kidney stone risk varied by education level, possibly reflecting differences in dietary habits, health awareness, and lifestyle behaviors. Hypertension status also showed a borderline interaction effect (*p* = 0.05), indicating a potential modifying role, though the results should be interpreted cautiously (Figure 4).

### 3.4 ROC curve analysis

**Figure 5:**
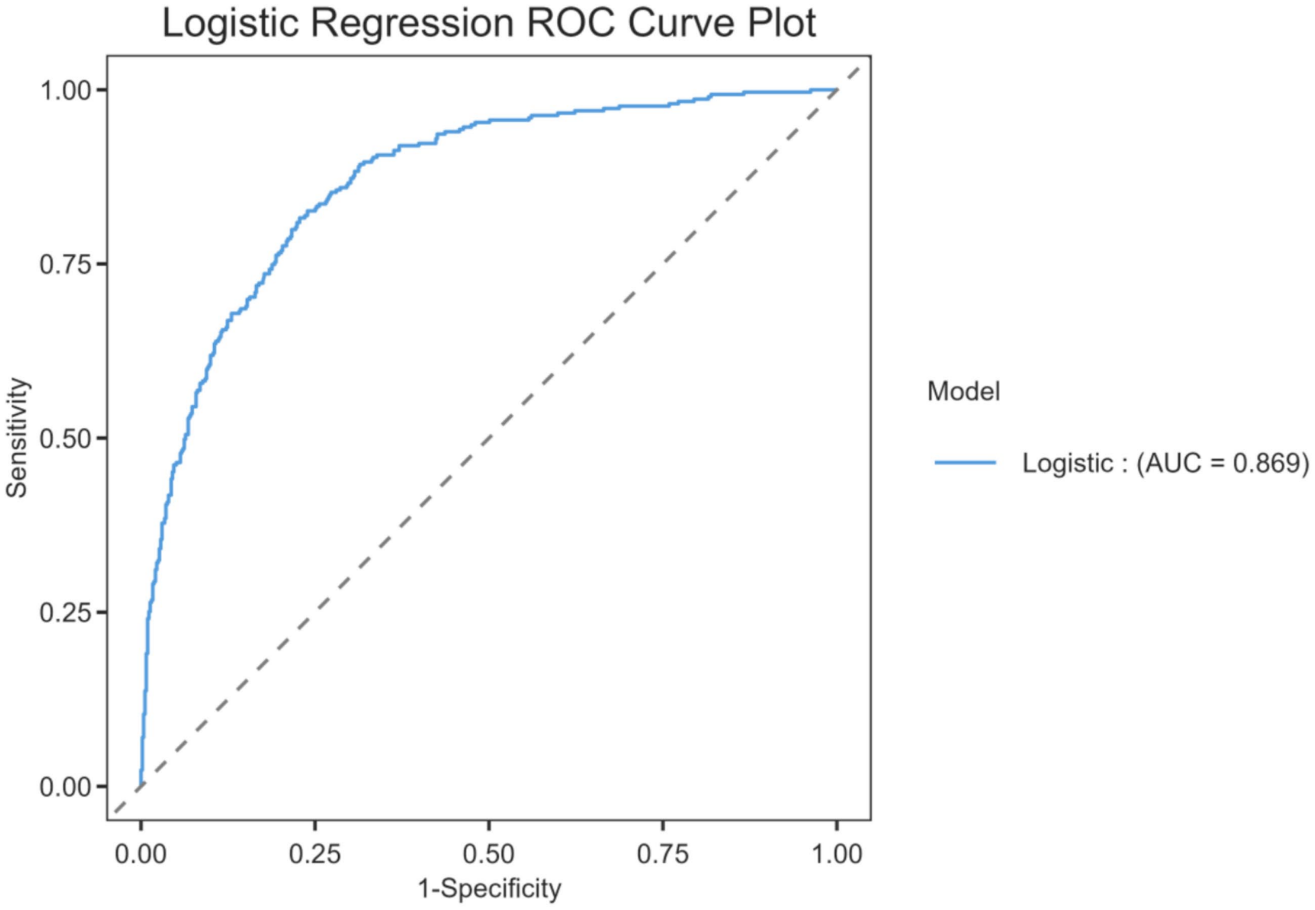
Receiver operating characteristic (ROC) curve of the multivariable logistic regression model for predicting kidney stone risk.

The predictive performance of the multivariable logistic regression model was evaluated using the ROC curve. The model achieved an area under the curve (AUC) of 0.869, indicating good discrimination between stone formers and non-stone controls (Figure 5).

## 4 Discussion

This study investigated the relationship between multiple types of meat consumption and kidney stone risk in a Chinese population. Consistent with previous findings, pork consumption emerged as positively associated with kidney stone with patients reporting significantly higher intake compared with controls. In contrast, sea fish intake demonstrated a negative association. Although processed meat was initially associated with stone risk, this effect was attenuated after adjusting for pork consumption, indicating that pork may be the primary driver. Furthermore, comorbidities such as hypertension and diabetes independently increased kidney stone risk, whereas higher daily water intake was associated with lower odds of kidney stone. These results highlight the interplay between dietary patterns, chronic metabolic conditions, and hydration in shaping kidney stone susceptibility.

Consistent with previous evidence (8), pork consumption was associated with an increased risk of kidney stones, particularly in populations where pork is the predominant source of dietary protein, such as in China (13). Pork is rich in purines, saturated fats, and animal protein, all of which have been implicated in lithogenic metabolic processes (5, 14, 15). High intake of purine-rich foods elevates serum and urinary uric acid levels, contributing to the formation of uric acid stones and enhancing the crystallization of calcium oxalate through heterogeneous nucleation (5). Additionally, excessive consumption of animal protein leads to acid load, promoting urinary acidification and reducing citrate excretion—an important natural inhibitor of stone formation (16). Moreover, high protein intake increases urinary calcium excretion while decreasing urine pH, both of which further enhance the risk for calcium-based kidney stones (17).

In contrast, sea fish intake was associated with a reduced risk of kidney stones. Sea fish are rich in omega-3 polyunsaturated fatty acids (PUFAs), particularly EPA and DHA, which exhibit anti-inflammatory effects by suppressing arachidonic acid metabolism and reducing prostaglandin production. These mechanisms contribute to lower urinary calcium and oxalate excretion, thereby reducing the supersaturation of stone-forming salts (6, 18). Although certain oily fish are high in purines, the protective effect of omega-3 fatty acids may outweigh potential risks, in line with prior evidence that dietary purines have a limited impact on uric acid levels compared with genetic factors (15, 19, 20).

Processed meat intake was initially associated with kidney stone risk, but this association was no longer significant after adjusting for pork consumption, reflecting the fact that many processed meats in China are pork-based (21). This suggests that the observed association may largely reflect pork consumption rather than processed meat itself.

Beyond meat categories, these findings underscore the importance of considering both nutrient composition and cultural dietary patterns when evaluating kidney stone risk. While Western studies often emphasize beef or animal protein as the major contributors, pork predominates in China and may exert stronger effects in this context. The differential association between pork and sea fish highlights the complex interplay of pro-lithogenic (purines, saturated fats, animal protein) and protective (omega-3 PUFAs, antioxidant compounds) dietary factors. These results also align with mechanistic insights from metabolic studies showing that diets high in animal protein increase urinary calcium and uric acid, whereas diets enriched with omega-3 fatty acids may counteract renal oxidative stress and inflammation.

Our study further demonstrates the utility of a simplified FFQ, which allowed for efficient data collection in a large sample while maintaining validity, consistent with recommendations for dietary assessment tools in epidemiological research (22, 23).

Several limitations should be acknowledged. First, the cross-sectional design precludes assessment of temporality and causality, and reverse causation is possible because participants with kidney stones may have modified their diet after diagnosis. Second, dietary intake was self-reported and therefore subject to recall and reporting bias. The simplified FFQ assessed consumption frequency but did not collect portion size, total energy intake, cooking methods, sodium intake, or detailed nutrient intake. Third, the broad classification of fish as sea fish or freshwater fish may have resulted in exposure misclassification. Such misclassification may attenuate associations toward the null, although its direction cannot be determined with certainty. Fourth, the relatively high exclusion rate in the online survey may have introduced selection bias and limited the generalizability of the online sample. The excluded questionnaires contained missing, inconsistent, or unreliable information and therefore could not support a valid comparison with the included participants. Fifth, stone composition was not collected, preventing separate analyses of calcium oxalate, uric acid, and other stone subtypes. Finally, residual confounding cannot be excluded, and differences between online and hospital-based recruitment may have introduced additional population heterogeneity.

Future prospective cohort studies and intervention trials are warranted to confirm the causal role of specific meat types, especially pork and fish, in stone pathogenesis. Moreover, integration of dietary assessment with metabolomics and genetic data may help to clarify biological pathways linking dietary components to kidney stone formation and identify individuals most susceptible to diet-induced risk. From a translational perspective, the dietary associations observed in this study may help inform patient education and lifestyle counseling, although they should not be interpreted as sufficient evidence for specific causal dietary prescriptions. Mobile health applications may provide a practical means of reinforcing evidence-based recommendations regarding fluid intake, diet, and physical activity and of supporting long-term self-management among patients with kidney stone disease (24).

## 5 Conclusion

In this cross-sectional study of a Chinese population, pork consumption was positively associated with kidney stone status, while sea fish intake was inversely associated. Hypertension and diabetes were confirmed positively associated with kidney stone status, and higher daily water intake was inversely associated. These findings provide population-specific evidence supporting the role of dietary meat patterns in kidney stone risk and highlight potential dietary strategies for prevention in China.

## Data Availability

The original contributions presented in the study are included in the article/Supplementary material, further inquiries can be directed to the corresponding authors.

## Footnotes

1
https://www.wjx.cn
